# Myocardial Injury after Surgery Is a Risk Factor for Weaning Failure from Mechanical Ventilation in Critical Patients Undergoing Major Abdominal Surgery

**DOI:** 10.1371/journal.pone.0113410

**Published:** 2014-11-19

**Authors:** Shu Li, You-zhong An, Jing-yi Ren, Feng-xue Zhu, Hong Chen

**Affiliations:** 1 Department of Critical Care Medicine, Peking University People's Hospital, Beijing, China; 2 Department of Cardiology, Peking University People's Hospital, Beijing, China; University of Louisville, United States of America

## Abstract

**Background:**

Myocardial injury after noncardiac surgery (MINS) is a newly proposed concept that is common among adults undergoing noncardiac surgery and associated with substantial mortality. We analyzed whether MINS was a risk factor for weaning failure in critical patients who underwent major abdominal surgery.

**Methods:**

This retrospective study was conducted in the Department of Critical Care Medicine of Peking University People's Hospital. The subjects were all critically ill patients who underwent major abdominal surgery between January 2011 and December 2013. Clinical and laboratory parameters during the perioperative period were investigated. Backward stepwise regression analysis was performed to evaluate MINS relative to the rate of weaning failure. Age, hypertension, chronic renal disease, left ventricular ejection fraction before surgery, Acute Physiologic and Chronic Health Evaluation II score, pleural effusion, pneumonia, acute kidney injury, duration of mechanical ventilation before weaning and the level of albumin after surgery were treated as independent variables.

**Results:**

This study included 381 patients, of whom 274 were successfully weaned. MINS was observed in 42.0% of the patients. The MINS incidence was significantly higher in patients who failed to be weaned compared to patients who were successfully weaned (56.1% versus 36.5%; *P*<0.001). Independent predictive factors of weaning failure were MINS, age, lower left ventricular ejection fraction before surgery and lower serum albumin level after surgery. The MINS odds ratio was 4.098 (95% confidence interval, 1.07 to 15.6; *P* = 0.04). The patients who were successfully weaned had shorter hospital stay lengths and a higher survival rate than those who failed to be weaned.

**Conclusion:**

MINS is a risk factor for weaning failure from mechanical ventilation in critical patients who have undergone major abdominal surgery, independent of age, lower left ventricular ejection fraction before surgery and lower serum albumin levels after surgery.

## Introduction

Prolonged mechanical ventilation has been associated with significant morbidity and mortality. Therefore, weaning should be considered as early as possible during the course of ventilation [1]. Postoperative patients are generally able to resume spontaneous ventilation as soon as they have recovered from anesthesia; therefore, the mode of ventilation should have little impact on the decision to extubate these patients. However, approximately 3 to 6% of patients admitted to the adult intensive care unit (ICU) require prolonged mechanical ventilation (cumulative duration of 24 hours) [2]–[3]. The rate of weaning failure is 5 to 8% in all surgical patients [4]–[5]. However, in patients who require prolonged mechanical ventilation, the rate of weaning failure is greater than 10%, and mortality and complication rates are also increased compared with other patients [6]–[8]. Unfortunately, the criteria and strategies for their successful weaning have been unclear [2]. Parameters of respiratory mechanics and oxygenation have commonly been used to wean patients with chronic obstructive pulmonary disease and other pulmonary disorders from long-term mechanical ventilation. However, these parameters are not sufficient for patients undergoing abdominal surgery whose pulmonary function is relatively normal.

Acute heart dysfunction or failure is another primary cause of weaning failure, largely due to acute myocardial ischemia during the perioperative period [9]. However, emerging evidence has suggested that many patients sustain myocardial injury during the perioperative period that does not satisfy the diagnostic criteria for myocardial infarction [10]. There have been only a limited number of studies that have reliably evaluated the incidence of myocardial injury after surgery, not to mention the effects of myocardial injury on weaning. Myocardial injury after noncardiac surgery (MINS) was recently proposed as a new concept [11]. The definition of MINS is broader than that of myocardial infarction because it includes not only the latter but also other prognostically relevant perioperative myocardial injuries due to ischemia. Evaluating patients for the presence of MINS, rather than myocardial infarction alone, could help physicians to avoid missing the majority of patients who develop prognostically relevant perioperative myocardial injury.

Therefore, we investigated the relationships of MINS and other perioperative parameters with the incidence of weaning failure in a cohort of critical patients who underwent major abdominal surgery. The aim of this study was to determine whether MINS and these parameters serve as risk factors of weaning failure.

## Materials and Methods

### Study design and patients

This study retrospectively analyzed patients who underwent abdominal surgery in the Department of Critical Care Medicine of Peking University People's Hospital between January 2011 and December 2013. All of the participants provided written informed consent. This study was approved by the Institution of Human Subjects Committee at Peking University People's Hospital. Critically ill patients were selected as the study subjects, according to the following inclusion and exclusion criteria.

#### Inclusion criteria

Patients meeting all of the following criteria were included in this study: (1) age >18 years old; (2) Acute Physiological and Chronic Health Evaluation II (APACHE II) score >8; (3) underwent endotracheal intubation and mechanical ventilation for abdominal surgery under general anesthesia; and (4) cumulative duration of postoperative mechanical ventilation >24 h. These criteria imply that patients who were not extubated within 24 hours as well as those who had one or more unsuccessful extubation attempts that resulted in an accumulated duration of at least 24 hours of mechanical ventilation were included.

#### Exclusion criteria

Patients who met the following criteria were excluded from this study: (1) patients who died or discontinued mechanical ventilation due to other reasons before weaning, (2) pregnant or nursing women, (3) patients who would underwent sequential noninvasive mechanical ventilation after weaning as planned, (4) patients who underwent tracheotomy, and (5) patients whose etiologies of elevated TnT were both ischemic and non-ischemic or could not be confirmed.

### Study procedures

#### Mechanical ventilation modes

According to the patient's condition, the treatment team responsible for the patient made decisions together regarding any adjustments of the mode and parameters of mechanical ventilation. The modes used for all of the patients included volume controlled ventilation, pressure controlled ventilation, and synchronized intermittent mandatory ventilation (pressure/volume), plus pressure support ventilation and pressure support ventilation.

#### Weaning procedure

The treatment team responsible for the patient evaluated daily whether the patient met the indications for weaning. If the patient met the indications, a spontaneous breathing trial (SBT) was conducted. For patients who succeeded in the trial, the tracheal cannula was immediately removed. Continuous electrocardiographic monitoring provided dynamic monitoring of arterial blood pressure, heart rate and finger oxygen saturation. Blood gas analysis was performed every 12 h. The patients were continuously observed for 48 h to determine whether weaning was successful.

#### Indications for weaning

When the patient essentially met the following indications for weaning [8], the treatment team responsible for the patient decided to initiate SBT.

Clinical evaluation criteria: forceful cough; no excessive airway secretion; the patient's underlying disease improved or began to improve.

Objective evaluation criteria: stable clinical condition; stable hemodynamic status; heart rate ≤140 beats/min; systolic blood pressure between 90 to 160 mm Hg; no or low-dose vasoactive drugs; stable metabolic status; modified oxygenation index ≥150 mm Hg; and positive end-expiratory pressure (PEEP) ≤8 cm H_2_O.

Respiratory function measurements: respiratory rate ≤35 times/min; pH >7.3; and arterial carbon dioxide pressure (PaCO_2_) of 35–50 mmHg.

#### SBT implementation methods

Every patient underwent one of the following methods. According to previous studies, there were no significant differences in sensitivity or specificity between the two SBT implementation methods in determining whether patients could be weaned from a breathing machine.

1. The tracheal cannula was connected to the breathing machine in pressure support mode. Inspiratory pressure ranged from 6 to 8 cm H_2_O, PEEP was 3 to 4 cm H_2_O, and the fraction of inspired O_2_ (FiO_2_) was 40%, lasting for 60–120 min.
2. The tracheal cannula was connected to an artificial nose. The oxygen flow was 3 to 5 l/min, lasting for 60–120 min.

#### SBT failure criteria

Patients who showed changes in mental status, including anxiety, restlessness, delirium, and somnolence, and/or who demonstrated the following criteria [8]:

Arterial oxygen pressure (PaO_2_) ≤50–60 mm Hg, PaCO_2_ ≥50 mmHg or increase >8 mm Hg; respiratory rate ≥35/min or increase ≥50%, heart rate ≥140 beats/min or ≥20% increase, systolic blood pressure ≥180 mm Hg or ≥20% increase, systolic blood pressure <90 mm Hg, or arrhythmia.

#### Weaning failure criteria

If the patients met any of the following criteria and required reintubation or if they died within 48 h after weaning, it was judged to be weaning failure [8]:

1. Respiratory rate ≥25 times/min for more than 2 h;
2. Heart rate ≥140 beats/min or persistent increase/decrease of ≥20%;
3. Patients developed evidence of respiratory muscle weakness or significant increase in breathing work, with oxygen saturation (SO_2_) <90%;
4. PaO_2_ <80 mm Hg, when FiO_2_ ≥50; or
5. Hypercapnia (PaCO_2_ >45 mm Hg or ≥20% increase compared with that before weaning) and pH <7.33.

### Grouping

The patients were divided into successful weaning and weaning failure groups according to whether they were successfully weaned or not.

### Observation indicators

Basic demographic characteristics of the patients included age, sex and body mass index (BMI).

The indicators for outcome evaluation included 28-day survival rate and the length of ICU stay.

Preoperative conditions included hypertension, diabetes, chronic renal disease, old myocardial infarction, chronic obstructive pulmonary disease, history of smoking, APACHE II score and preoperative left ventricular ejection fraction (LVEF).

Surgical data included intraoperative blood loss, duration of surgery and whether reoperation was performed within 24 h or not.

Postoperative data included pleural effusions, pneumonia, MINS, sepsis, acute kidney injury (AKI), duration of mechanical ventilation before weaning and Simplified Acute Physiology Score II (SAPS II) at the time of weaning.

Postoperative laboratory parameters included blood cardiac troponin I (cTnI), cTnT, N-terminal pro-brain natriuretic peptide (NT pro-BNP), calcium, magnesium, serum creatinine (SCr), hemoglobin (Hb) and albumin (Alb) levels.

Laboratory tests were routinely performed for all patients as indicated below. TnT and TnI were measured every 6 hours during the first 12 hours, every 24 hours during the following 3 days, and twice weekly until the patients were discharged from the ICU. These measurements were also obtained at the time of weaning and assessed more frequently if necessitated by additional conditions. NT pro-BNP, calcium, magnesium, SCr, Hb and Alb levels were measured daily, whereas urine volume was record by a nurse every 2 hours until the patient was discharged from the ICU.

### Definition of MINS

The definition used was prognostically relevant myocardial injury due to ischemia that occurred during or within 30 days after noncardiac surgery [11]. The diagnostic criterion for MINS was any peak TnT of 0.03 ng/ml or greater that was judged as resulting from myocardial ischemia. Each elevated TnT was evaluated to ensure an ischemic etiology.

### Definition of AKI

AKI was defined as a ≥0.3 mg/dl (≥26.5 mol/l) increase in SCr within 48 hours; or an increase in SCr of ≥1.5 times baseline, which is known or presumed to have occurred within the prior 7 days; or urine volume <0.5 ml/kg/h for 6 hours [12].

### Statistical analysis

SAS statistical software (SAS Institute Inc., Cary, NC, USA), version 9.1, was used for the statistical analyses. Measurement data are expressed as the means ± standard deviations or the medians (25th to 75th percentile). Enumeration data are expressed as absolute frequencies and percentages. For inter-group comparisons of basic demographic characteristics and clinical indicators, Student's *t* test was used for normally distributed measurement data, and the rank sum test was used for non-normally distributed measurement data. The χ^2^ test was used for comparisons of rates. Binary logistic regression was used for multivariate analysis. All of the tests were conducted at a level of significance of α = 0.05.

## Results

### Baseline clinical and demographic characteristics

**Figure 1:**
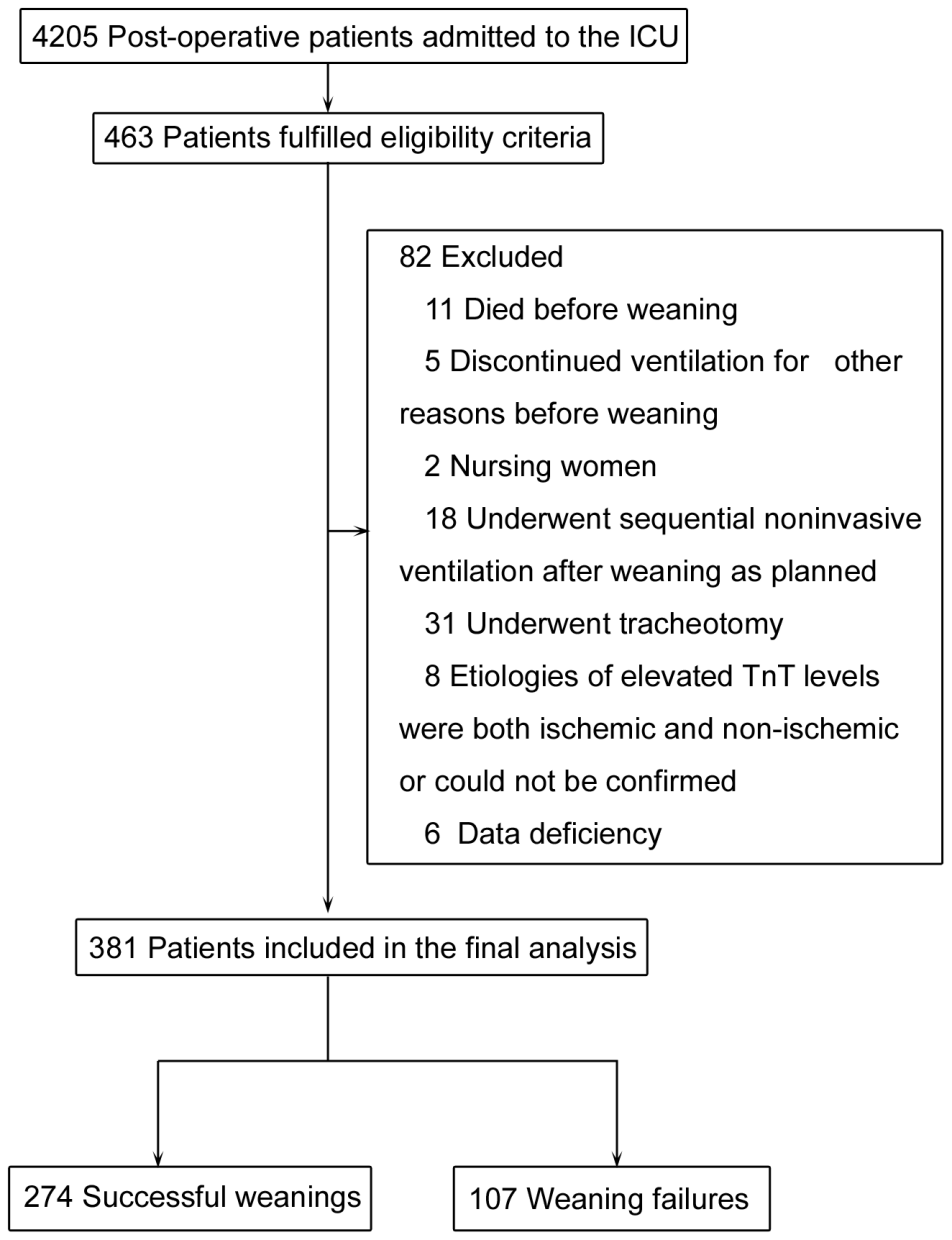
Patient flow chart.

Between January 2011 and December 2013, 4,205 patients were treated in the Department of Critical Care Medicine of Peking University People's Hospital. Among these patients, 381 were selected and included in this study. A flowchart of patient inclusion is shown in Figure 1. General patient information is shown in Table 1.

**Table 1:** Patient Demographic and Clinical Characteristics.

Characteristic	Value
Male sex, n (%)	212 (55.6)
Mean age (years)	63.8±16.5
Mean BMI (kg/m^2^)	23.4±4.4
Mean length of stay in ICU (days)	6 (4 to 12)
Duration of mechanical ventilation before weaning (hours)	66 (39 to 144)

Data are presented as medians (25th to 75th percentile) or numbers (% of patients). BMI, body mass index; ICU, intensive care unit.

### Comparison of clinical characteristics at admission to the ICU

According to whether they were successfully weaned or not, the patients were divided into successful weaning and weaning failure groups. The statistical results showed that patients in the successful weaning group were younger with a lower rate of hypertension, fewer chronic renal diseases, higher preoperative LVEF and lower APACHE II score on admission to the ICU than patients in the weaning failure group. These differences were statistically significant (*P*<0.05). Detailed results are shown in Table 2.

**Table 2:** In-ICU Variables.

Variables	Success (n = 274)	Failure (n = 107)	*P* value
Mean age (years)	60.95±16.84	70.3±13.76	<0.001
Male sex, n (%)	154 (56.2)	58 (54.2)	0.732
Mean BMI (kg/m^2^)	23.22±4.20	23.72±4.96	0.487
Complications, n (%)			
Hypertension	80 (29.2)	64 (59.8)	<0.001
Diabetes mellitus	28 (10.2)	18 (16.8)	0.082
Chronic renal disease	6 (2.19)	14 (13.1)	<0.001
Previous myocardial infarction	8 (2.92)	6 (5.61)	0.230
Chronic obstructive pulmonary disease	12 (4.38)	5 (4.67)	0.545
Smoking	30 (10.9)	8 (7.48)	0.348
LVEF (%)	68.9 (65 to 72)	65.6(59.3 to 70.7)	0.039
APACHE II	18 (14 to 21)	25 (20 to 29)	0.027

Data are presented as medians (25th to 75th percentile) or numbers (% of patients). BMI, body mass index; LVEF, left ventricular ejection fraction; APACHE II, Acute Physiology and Chronic Health Evaluation II.

### Comparison of intraoperative and postoperative situations

The statistical results showed that the patients in the successful weaning group had lower incidences of postoperative pneumonia, pleural effusion, MINS and AKI and a shorter duration of mechanical ventilation before weaning than the patients in the weaning failure group. These differences were statistically significant (*P*<0.05). Detailed results are shown in Table 3.

**Table 3:** Intraoperative and Postoperative Variables.

Variables	Success (n = 274)	Failure (n = 107)	*P* value
Operative blood loss (ml)	650 (200 to 1950)	500 (150 to 1450)	0.200
Duration of surgery (hours)	4.5 (3 to 6.5)	3.5 (2.5 to 5)	0.079
Reoperation in 24 hours, n (%)	10 (3.65)	4 (3.74)	0.587
Pleural effusion, n (%)	86 (31.4)	46 (43.0)	0.041
Pneumonia, n (%)	70 (25.5)	48 (44.9)	<0.001
MINS, n (%)	100 (36.5)	60 (56.1)	<0.001
Sepsis, n (%)	88 (32.1)	46 (43.0)	0.056
Acute kidney injury, n (%)	70 (25.5)	50 (46.7)	<0.001
Duration of mechanical ventilation before weaning (hours)	46.5 (42.5 to 37.5)	75.5 (14 to 184)	0.048

Data are presented as medians (25th to 75th percentile) or numbers (% of patients). MINS, myocardial injury after noncardiac surgery.

### Comparison of laboratory indicators and disease condition scores at the time of weaning

The statistical results showed that patients in the successful weaning group had higher blood albumin and lower SAPS II scores than patients in the weaning failure group. These differences were statistically significant (*P*<0.05). Detailed results are shown in Table 4.

**Table 4:** Variables before Weaning.

Variables	Success (n = 274)	Failure (n = 107)	*P* value
cTnI (ng/ml)	0.020 (0.007 to 0.035)	0.034 (0.009 to 0.067)	0.319
NT pro-BNP (pg/ml)	217 (73 to 405)	247 (108 to 424)	0.908
Ca^2+^ (mmol/l)	1.15 (1.11 to 1.19)	1.135 (1.09 to 1.18)	0.222
Mg^2+^ (mmol/l)	0.55 (0.49 to 0.63)	0.55 (0.48 to 0.64)	0.613
SCr (µmol/l)	61.5 (49 to 85.5)	69 (57.5 to 118)	0.116
Hb (g/l)	97.44±14.11	96.32±13.58	0.617
Alb (g/l)	33.94±4.17	30.36±5.01	<0.001
SAPS II	24 (18 to 31)	37 (23 to 52)	0.016

Data are presented as medians (25th to 75th percentile) or numbers (% of patients). cTnI, cardiac troponin I; NT pro-BNP, N-terminal pro-brain natriuretic peptide; SCr, serum creatinine; Hb, hemoglobin; Alb, albumin; SAPS II, Simplified Acute Physiology Score II.

### Outcome comparison between the successful weaning and weaning failure groups

In the successful weaning group, 5 patients died with 28-day mortality of 1.82%; in the weaning failure group, 41 patients died with 28-day mortality of 38.32%. This difference was statistically significant (χ^2^ = 91.85, *P*<0.001). The median length of ICU stay was 6 days in the successful weaning group and 9 days in the weaning failure group. This difference was statistically significant (t = −4.179, *P*<0.001).

### Risk factors for weaning failure

In accordance with the above results, binary nonconditional logistic regression analysis was performed using the backward LR method, with parameters that showed statistically significant differences between the two groups in the previous analysis as the independent variables and weaning success as the dependent variable. Age, LVEF and blood albumin were the continuous variables, while postoperative occurrence of MINS was a categorical variable (with MINS  = 1, without MINS  = 0). The results showed that advanced age, low preoperative LVEF, postoperative occurrence of MINS and low blood albumin at the time of weaning were independent risk factors influencing the weaning success rate. Of all the MINS patients, 79.4% (127/160) were diagnosed within 24 hours after surgery, 97.5% (156/160) were diagnosed within 72 hours after surgery, and 98.6% (158/160) were diagnosed within the first week. Regarding the 2 cases who were not diagnosed within the first week, one was diagnosed on the 8^th^ day, whereas the other patient was diagnosed on the 9^th^ day after surgery. Detailed results are shown in Table 5.

**Table 5:** Multiple Regression.

Variables	*P* value	Odds ratio (95% CI)
Age	0.006	1.100 (1.028–1.176)
LVEF before surgery	0.008	0.861 (0.772–0.962)
MINS	0.040	4.098 (1.070–15.63)
Serum albumin level	0.043	0.812 (0.664–0.993)

LVEF, left ventricular ejection fraction; MINS, myocardial injury after noncardiac surgery.

## Discussion

Postoperative occurrence of MINS is an important factor influencing cardiovascular function. In our study, regression analysis showed that MINS was an independent risk factor for weaning failure with a greater than 4-fold increased risk of failure. Acute myocardial ischemia during the perioperative period is not always due to local stenosis or spasm of the coronary artery. After surgery, patients are in a state of stress because of the pain after recovery from anesthesia, the stress of the surgery itself and intolerance to the tracheal cannula, all of which contribute to a significant increase in blood catecholamines. This increase leads to an increased cardiac load and a significant increase in the risk of myocardial ischemia [11]. In addition, intraoperative and postoperative hemodynamic instability is the main cause of myocardial hypoperfusion, ischemia, hypoxia and particularly myocardial injury. If patients have concurrent coronary heart disease and chronic heart disease, perioperative hemodynamic instability and systemic ischemia and hypoxia are more likely to cause cardiac dysfunction during weaning, ultimately resulting in extubation failure [13]–[14]. Following abdominal surgery, factors such as postoperative abdominal pain, the use of abdominal bandages, long-term immobilization, intra-abdominal infections and diaphragmatic dysfunction often influence intrathoracic pressure and lung volume. The effects of these factors on respiratory function and hemodynamics cannot be ignored.

Potential insufficient cardiovascular reserve and compensatory capacity before surgery are important factors that influence postoperative weaning failure. In our study, multivariate regression analysis showed that advanced age and low preoperative LVEF were independent risk factors for weaning failure. In addition, patients in the weaning failure group had a higher proportion of concomitant underlying high-risk cardiovascular diseases, with higher rates of hypertension and chronic renal disease than the patients in the successful weaning group. These are all important factors that can lead to cardiovascular dysfunction and decreased compensation.

It has been estimated that the global population will show a trend of accelerated aging over the next 20 years, and the elderly population requiring surgery will be more than 4 times larger than the population in other age groups [15]. Increased age leads to a higher rate of perioperative complications [16]. Among the various perioperative complications in elderly patients, cardiovascular diseases have the highest incidence. In a study of patients between 75 and 84 years old, 19% of men and 12% of women had one or more cardiovascular diseases of varying severity [17]. Thus, these patients were more likely to have insufficient cardiac reserves before surgery. In our study, echocardiography also showed that, although patients in both groups had LVEF values within the normal range, patients in the successful weaning group had higher LVEF values than those in the weaning failure group.

In this study, patients with definite diagnoses of underlying cardiovascular diseases were few in number. The patients in the successful weaning group had a slightly higher rate of cardiovascular diseases than patients in the weaning failure group. However, perhaps due to the small sample size, the difference was not statistically significant. This finding does suggest, however, that it is not sufficient to focus only on the presence of documented underlying cardiovascular diseases during preoperative evaluations. The evaluation and improvement of potentially insufficient cardiovascular reserve and compensation are of great importance for improving the postoperative weaning success rate.

Poor nutritional status has consistently been recognized as one of the factors influencing weaning difficulty. Theoretically, patients with poor nutritional status are in negative nitrogen balance after surgery, which can induce disorders and dysfunctions of respiratory muscle strength and can even result in pulmonary interstitial edema. Patients with a low body mass index can develop respiratory drive suppression, decreased muscle volume and weaning difficulty [18]. The results of our study also showed that after abdominal surgery, critically ill patients who were successfully weaned had significantly higher blood albumin levels at the time of weaning than patients who failed weaning. Hypoalbuminemia was an independent risk factor for weaning failure. Despite this observation, few studies have investigated the associations between low albumin levels and weaning failure. While low albumin levels might suggest poor nutritional status, they can also be related to the surgical procedure. Further, patients usually cannot eat a normal diet after abdominal surgery. Low albumin itself can be corrected through treatment in a relatively short time. However, further studies are required to investigate in greater detail whether hypoalbuminemia is a reflection of poor nutritional status, of a metabolic disorder or of other issues.

Our study analyzed the outcomes of critically ill patients after abdominal surgery. The results showed that patients who were successfully weaned had shorter ICU stays and overall durations of mechanical ventilation than patients who failed weaning. Furthermore, there was a significant difference in 28-day mortality between the two groups. There are many factors that influence the outcomes of critically ill patients after abdominal surgery. Because this study was a retrospective case analysis, it was impossible to determine whether weaning was a risk factor for poor outcomes due to the limited case data. Our available results were similar to the results from other domestic and overseas studies. Other studies have indicated that after statistical adjustment for disease conditions, underlying diseases and age, weaning failure remains an independent risk factor for predicting in-hospital mortality [19]. Additionally, weaning failure exacerbates the deterioration of underlying diseases and complications, such as aspiration, atelectasis and even pneumonia [20]. These complications, combined with the direct complications caused by reintubation and the instability of the disease condition between extubation and reintubation, contribute to the increased mortality of patients who fail weaning. Prolonged mechanical ventilation also causes side effects that can contribute to a poor outcome. For example, the incidence of nosocomial pneumonia increases after prolonged mechanical ventilation. The development of these complications is also an independent risk factor for inpatient mortality [21]–[22]. Thus, exploring the methods and means needed to improve the postoperative weaning success rate is extremely important for improving patient outcomes and reducing perioperative mortality.

The present study has its strengths and limitations. This is the first study demonstrating that MINS is an independent risk factor for weaning failure in critical patients who have undergone major abdominal surgery. Our results also indicate that potentially insufficient cardiovascular reserve and compensatory capacity before surgery and poor nutritional status are important factors that potentially influence postoperative weaning failure. Our study also has several limitations. First, it is a single center study. Although numerous confounding factors could influence weaning from ventilation, we only included a limited number of factors given the small sample size. Second, this study is retrospective, and medical history and diagnosis data could alter the accuracy of our results.

In conclusion, MINS, as diagnosed based on any peak TnT of 0.03 ng/ml or greater, was a risk factor for weaning failure in critical patients who underwent major abdominal surgery, independent of age, lower LVEF before surgery and lower serum albumin level after surgery. For patients with underlying heart disease and MINS, it is therefore necessary to further evaluate whether their heart function can tolerate the load that results from weaning, as well as the current indications for weaning. Advanced age and low blood albumin at the time of weaning were also risk factors influencing the weaning success rate. For these patients, the indications and the timing of weaning should be strictly monitored to improve the weaning success rate and patient outcomes.
